# Lung molecular and histological changes in type 2 diabetic rats and its improvement by high-intensity interval training

**DOI:** 10.1186/s12890-024-02840-1

**Published:** 2024-01-17

**Authors:** Mohammad Amin Rajizadeh, Kayvan Khoramipour, Siyavash Joukar, Fatemeh Darvishzadeh-Mahani, Maryam Iranpour, Mohammad Abbas Bejeshk, Maryam Doustaki Zaboli

**Affiliations:** 1 Physiology Research Center, Institute of Neuropharmacology, Kerman University of Medical Sciences, Kerman, Iran; 2 Cardiovascular Research Center, Institute of Basic and Clinical Physiology Sciences, Kerman University of Medical Sciences, Kerman, Iran; 3 Department of Physiology and Pharmacology, Afzalipour Medical Faculty, Kerman University of Medical Sciences, Kerman, Iran; 4 Neuroscience Research Center, Institute of Neuropharmacology, Kerman University of Medical Sciences, Kerman, Iran; 5 Pathology and Stem Cell Research Center, Department of Pathology, Afzalipour Medical Faculty, Kerman University of Medical Sciences, Kerman, Iran

**Keywords:** Type 2 diabetes, HIIT, Oxidative stress, Inflammation, Apoptosis, Pulmonary surfactants

## Abstract

**Background:**

Type 2 diabetes (T2D) leads to serious respiratory problems. This study investigated the effectiveness of high-intensity interval training (HIIT) on T2D-induced lung injuries at histopathological and molecular levels.

**Methods:**

Forty-eight male Wistar rats were randomly allocated into control (CTL), Diabetes (Db), exercise (Ex), and Diabetes + exercise (Db + Ex) groups. T2D was induced by a high-fat diet plus (35 mg/kg) of streptozotocin (STZ) administration. Rats in Ex and Db + Ex performed HIIT for eight weeks. Tumor necrosis factor-alpha (TNFα), Interleukin 10 (IL-10), BAX, Bcl2, Lecithin, Sphingomyelin (SPM) and Surfactant protein D (SPD) levels were measured in the bronchoalveolar lavage fluid (BALF) and malondialdehyde (MDA) and total antioxidant capacity (TAC) levels were measured in lung tissue. Lung histopathological alterations were assessed by using H&E and trichrome mason staining.

**Results:**

Diabetes was significantly associated with imbalance in pro/anti-inflammatory, pro/anti-apoptosis and redox systems, and reduced the SPD, lecithin sphingomyelin and alveolar number. Performing HIIT by diabetic animals increased Bcl2 (*P* < 0.05) and IL10 (*P* < 0.01) levels as well as surfactants components and TAC (*P* < 0.05) but decreased fasting blood glucose (*P* < 0.001), TNFα (*P* < 0.05), BAX (*P* < 0.05) and BAX/Bcl2 (*P* < 0.001) levels as well as MDA (*P* < 0.01) and MDA/TAC (*P* < 0.01) compared to the diabetic group. Furthermore, lung injury and fibrosis scores were increased by T2D and recovered in presence of HIIT.

**Conclusion:**

These findings suggested that the attenuating effect of HIIT on diabetic lung injury mediated by reducing blood sugar, inflammation, oxidative stress, and apoptosis as well as improving pulmonary surfactants components.

**Graphical Abstract:**

Type 2 diabetes increased inflammation, oxidative stress and apoptosis and reduced pulmonary surfactants , while high intensity training improved these negative effects

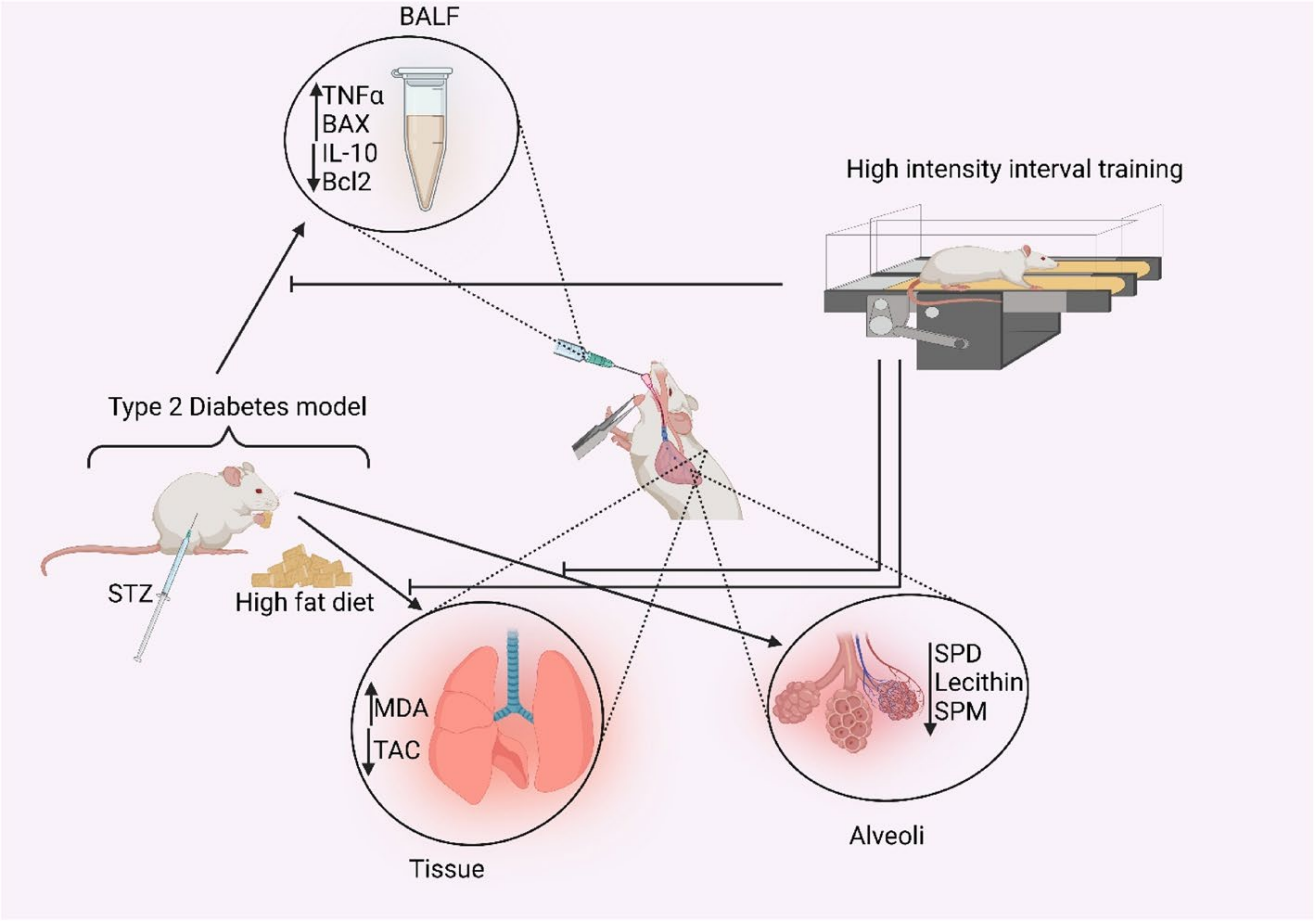

**Supplementary Information:**

The online version contains supplementary material available at 10.1186/s12890-024-02840-1.

## Introduction

Diabetes is a metabolic disorder that is associated with continuous hyperglycemia and abnormal metabolism of carbohydrates, proteins, and lipids, caused by insufficient insulin secretion, and decreased tissue sensitivity to insulin [1]. According to the World Health Organization (WHO) in 2016, the prevalence of diabetes was 171 million people in 2000, and it is projected to reach 366 million people by 2030. Type 2 diabetes (T2D) accounts for 90–95% of all cases, where the body becomes resistant to insulin, and insulin cannot enter cells [2]. Hyperglycemia disrupts the oxygen balance and cellular regeneration, increasing the gene expression of cytokines, and inflammation, and inducing apoptosis [3].

The lung is one of the organs that are damaged in T2D. NADH/NAD imbalance increases the production of reactive oxygen species (ROS), oxidative stress, and cell death in diabetic lungs [4]. Mechanisms that cause lung dysfunction in diabetics include oxidative stress [5], microangiopathy of capillaries of alveoli and lung arteries [6], changes in lung elastin and collagen [7], and surfactant dysfunction [8]. Surfactant, secreted in the alveolar space, is a lipoprotein complex produced by type 2 alveolar cells [9]. Hyperglycemia disrupts the expression of surfactant proteins genes [8]. Diabetes and insulin resistance decrease the levels of antioxidants such as catalase (CAT), glutathione (GSH), and superoxide dismutase (SOD) [10]. T2D leads to an increase in inflammatory cytokines such as TNF-α and IL-6 and a decrease in some anti-inflammatory cytokines such as IL-10 [5, 11].

Exercise training has been accepted as a non-pharmacological therapy to improve health conditions [12–14]. It has been proven that exercise training protects diabetic patients against oxidative stress by increasing the amount of antioxidant enzymes [15, 16]. An experimental study demonstrated that moderate and regular exercise training reduces CRP, IL-6, TNF-α and increases adiponectin levels and IL-10 [17].In general, exercise has anti-inflammatory effects especially in diabetes [18]. Another study has shown that aerobic exercise plays a protective role in lipopolysaccharide (LPS)-induced acute lung injury [19].In addition, it has been shown that exercise can protect lungs against methotrexate-induced lung injury [20]. Furthermore, the beneficial effects of aerobic exercise on blood sugar control and diabetes complications increase with exercise intensity, and more adaptation is achieved with high-intensity interval training (HIIT) [21].

In this study, we used of rats as an animal model for T2D induction. The rat as an experimental animal model of human disease offers various favorable circumstances and advantages over the mouse and different species [22, 23]. Rat is extensively used as a suitable animal model for understanding the metabolic profile and pathology involved in different stages of type 2 diabetes [24].

Studies related to the effects of exercise on the diabetic lung are incomplete and, we are facing to lack of information regarding the impact of HIIT exercise on the diabetic lung. Therefore, considering the negative effect of T2D on lung tissue and the positive effects of appropriate exercise on health stability, in the present study the impact of a specific type of exercise i.e., HIIT was investigated on some inflammatory, oxidative, apoptotic, surfactant, and histopathological indices of the lungs of diabetic rats.

## Method

### Animal care

**Fig. 1:**
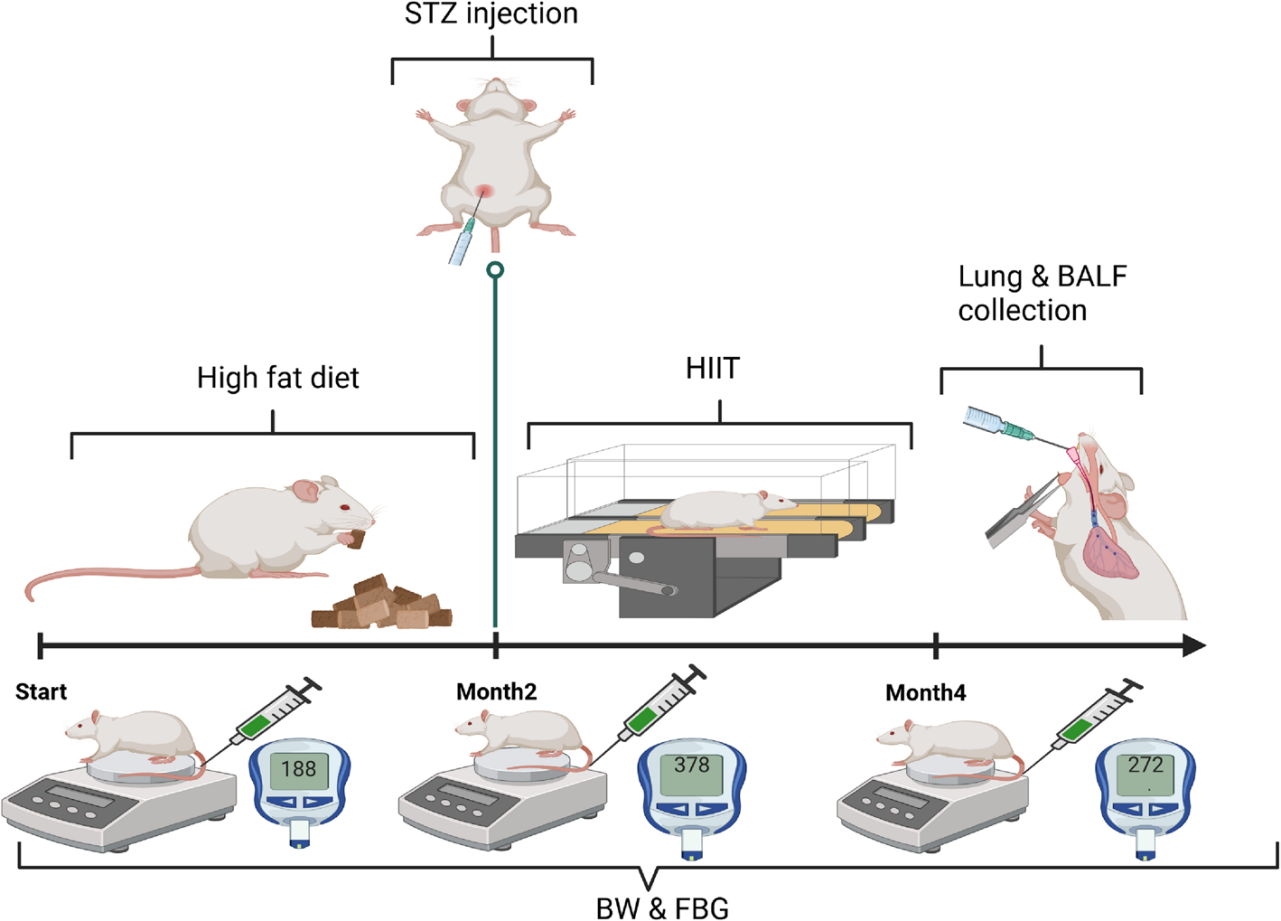
Time-line of the experiment. BW; Body weight, FBG; Fasting blood glucose, HIIT; High intensity of training

Twenty-eight eight-week-old male Wistar rats with an average weight of 200 g were purchased from the animal farm of Kerman University of Medical Sciences (KUMS) and kept them in special polycarbonate cages at 23 ± 2 °C and a 12:12 dark–light cycle. Throughout the experiment, all animals had *ad libitum* access to food and water. All experimental protocols were approved by ethics committee of Kerman university of medical sciences (Ethics approval code: IR.KMU.AH.REC.1400.173). For reducing suffering and distress of animals, their cages were cleaned every day. During the study to reduce the pain of the animals, we did not use electric shocks during exercise, and at the end of the study, the animals were sacrificed with a high dose of ketamine and xylazine, and we tried to do this process without pain. Also, Ethics approval all methods are reported in accordance with ARRIVE guidelines. After being acclimatized to the laboratory environment, the animals were randomly assigned to one of four groups (*n* = 7 in each group): control (CTL), type 2 diabetes (Db), exercise (Ex), and type 2 diabetes + exercise (Db + Ex). The Ex and Db + Ex groups performed 8 weeks of HIIT.A schematic representation for method stages was illustrated in Fig. 1.

### Induction of diabetes

Db + Ex and Db groups were fed a high-fat diet (HFD) for 2 months, as shown in Table 1. After this period, the animals fasted for 12 h and then received a single intraperitoneal injection of 35 mg/kg streptozotocin (STZ). Three days later, their fasting blood glucose (FBG) levels were measured using a glucometer. FBG levels were measured at three time points: before starting the intervention (month 0), after diabetes induction (2 months of HFD and STZ injection), and 48 h after the training period using an Accu-Chek glucometer (USA) [25, 26].

**Table 1:** A normal and high-fat (HFD) ingredients

Type of diet	Fat	Carbohydrate	Protein	Fiber	Minerals	Vitamins
**Normal die**	10%	70%	20%	50gr	50gr	3gr
**High fat diet**	60%	20%	20%	50gr	50gr	3gr

This table indicates the percentage of ingredients of regular and high fat diets. The fat percentage in HFD is higher than normal diet while the carbohydrate percentage is lower. The rest of ingredients are equal between two types of diets

### Exercise protocol

All of the animals were familiarized with a motorized treadmill prior to the experiments. They ran on the treadmill at a speed of 8 m/min with an incline of 0% for 10 min per day over the course of 5 consecutive days. Both the Ex and T2D + Ex groups performed an incremental running test to determine their maximum speed (Vmax). They ran for 2 min at a speed of 6 m/min speed, and every 2 min the speed was increased by 2 m/min until the rats were exhausted. The last min tolerated speed was considered Vmax. The HIIT protocol was carried out five times a week for 8 weeks by rats in Ex and T2D + Ex groups [30]. The rats’ Vmax was measured every 2 weeks, and the new Vmax was used to calculate relative speed for the next 2 weeks. This protocol was designed in our lab, and is referred to as the K1 protocol (Table 2) [25]. All ethical principles of exercise in animals were considered in this study. For example, the design of the treadmill was such that it was not possible to fall off the animal from treadmill and also, the rats did not breathe hard and did not get exhausted because at the beginning of the study based on VO2max, an exercise protocol designed for animals. In addition, the VO2max was measured during study period.

**Table 2:** High-intensity interval training (HIIT) protocol

Week	Slope	Frequency (Number/week)	Intervals (Number/session)	High-intensity interval duration (min)	Low-intensity interval duration (min)	High-intensity interval velocity (Vo2max%)	Low-intensity interval velocity (Vo2max%)	Total exercise time in a session (min)
1	0	5	4	2	1	80	50	12
2	0	5	4	2	1	85	50	12
3	0	5	6	2	1	85	50	18
4	0	5	6	2	1	90	50	18
5	0	5	8	2	1	90	50	24
6	0	5	8	2	1	95	50	24
7	0	5	10	2	1	95	50	30
8	0	5	10	2	1	100	50	30

This table represents the details of exercise protocol. The HIIT protocol incorporated alternating intervals of high and low intensity, determined by VO2max%. Intervals above 80% of VO2max% were designated as high intensity, while those below this threshold were classified as low intensity. This approach ensured a structured and effective exercise regimen for the rats throughout the training period

### Serum and tissue sampling

After 48 h since the last training session, the animals were anesthetized by intraperitoneal injection of ketamine (80 mg/kg) and xylazine (10 mg/kg). Blood samples were collected from the animal’s heart after 12 h of fasting, and right lung tissues were harvested and stored in freezer for molecular investigations. The blood samples were left at room temperature for 30 min and then centrifuged at 1000 g for 20 min at 4 °C was to measure BGF. The resulting serum samples were stored at a temperature of – 80 °C [27].

### BALF collection

After the sacrifice of the animal, bronchoalveolar lavage fluid (BALF) was collected from the left lung. Briefly, a median sternotomy was performed, the trachea was isolated. The right main bronchus was clamped and a catheter was inserted to the left main bronchus of the animal. Then 2.5 mL of normal saline was instilled into the bronchoalveolar space of the left lung and the instilled fluid was then harvested by aspiration into the syringe. The right lung was not washed to preserve it for histopathology and molecular evaluations. The BALF was immediately centrifuged (4 ◦C, 10 min, 1000 g), and the supernatant was used to measure the levels of cytokines and surfactant proteins and apoptotic factors [28, 29].

### ELISA

The levels of TNFα (Bio-techne Co, USA, Catalog No: DY510-05), IL-10 (Bio-techne Co, USA, Catalog No : R1000), SP-D (Elabscience Co, USA, Catalog No : E-EL-R0831), Lecithin (Sigma-Aldrich Co, USA, Catalog No : MAK049 ), SPM (Sigma-Aldrich Co, USA, Catalog No : MAK154), BAX (LSBio Co, USA, Catalog No : LS-F5064 ) and Bcl2 (LSBio Co, USA, Catalog No : LS-F4135) were assessed using ELISA in the BALF according to the manufacturer’s instructions of relevant kits protocol. Oxidative stress factors were measured in lung tissue. For this purpose, the lung tissues were homogenate through sonication and total proteins were assessed by Bradford method in homogenate samples. Malondialdehyde (MDA), as an index of lipid peroxidation, was estimated using the concentration of thiobarbituric acid reactive substances (TBARS) at 550 nm and Total antioxidant capacity (TAC) was determined by the ferric reducing ability of plasma (FRAP) assay at 593 nm [30] .

### Histopathology

The right lungs of animals were harvested and immersed in 10% formalin. After the paraffin molding of the tissues, 5 mm thick sections which were stained with hematoxylin and eosin (H&E) were prepared, and later examined microscopically by a pathologist who was blind to the animal groupings. Six fields of same area of the lung for each rat were analyzed [31, 32]. Also, Mason trichrome staining was done to measure tissue fibrosis score according to the Hübner et al. scoring system [33]. The slides were examined by a pathologist, who was blinded to the group of animals. H&E-stained slides were scored as: absent (0), minimal (1), mild (2), moderate (3), and severe (4) lesions for peribronchial inflammation, inflammatory cell infiltration, expansion of the alveolar interstitial space, enlargement of airway, destruction of septum of alveoli, congestion, and fibrosis. The total lung score was expressed as the sum of the scores for each parameter. Mean alveolar number (MAN) as an indicator for density of alveoli was calculated by MAN = AN/surface area (SA) (mm^2^) formula according to AN in each field of view and SA of the field [14, 34].

### Statistical analysis

The data are reported as a mean ± standard error of the mean (SEM). The Shapiro Wilk test was used for examining the normality of the data. One-way analysis of variance (ANOVA) followed by Tukey’s post hoc test were used for analysis of parametric data. The Kruskal-Wallis and Mann–Whitney test was utilized for analysis of lung injury and fibrosis data. The significance level was considered at *P* < 0.05.

## Results

### The effects of HIIT and T2D on FBG and body weight

The FBG assessed to confirm diabetes induction method and the effect of HIIT on its level. Fasting blood glucose was significantly increased after diabetes induction (2 months of high-fat diet and STZ injection) (month 2) compared with baseline (month 0) in Db and Db + Ex group (*P* < 0.001), with no significant difference between these groups. Performing HIIT significantly reduced FBG in Db + Ex group vs. Db group (*P* < 0.001) (Table 3). In other words, exercise was able to bring blood glucose levels closer to normal levels.

**Table 3:** FBG and BW (mean ± SD) before starting the intervention (month 0), after diabetes induction (2 months of high-fat diet and STZ injection) (month 2), and 48 h after the last training session (month 4) in all groups

Parameter	Group	Pre-test (Month 0)	Month 2 (After STZ injection)	Post-test (Month 4)	Percentage of changes at the end of study (%)
FBG (mg/dl)	CTL	102±8	98±9	105±12	2.9
	Db	88±9	378±7^**^	272±7^##^	67.6
	Ex	90±8	92±8	91±8	1.09
	Db+Ex	89±7	396±7^**^	212±10^##^	58
BW (gr)	CTL	214±18	285±14^*^	317±13	32.4
	Db	205±12	398±25^**^	307±15^#^	33.2
	Ex	211±9	283±11^*^	314±8	32.8
	Db+Ex	202±10	381±21^**^	319±21^#^	36.6

*FBG* Fasting blood glucose, *BW* Body weight, *CTL* Control, *Db* Type 2 diabetic (STZ injected), *Ex* Exercise only, and Db+Ex: Type 2 diabetic+ exercise

*P<0.05 and ** P<0.01 versus month 0

^#^P<0.05 and ^##^P<0.01 versus month 2 of corresponding groups

On the other hand, animals’ body weight significantly increased in Db and Db + Ex groups after diabetes induction (month 2) in rats. In addition, the weight was decreased in Db and Db + Ex groups, with more decrease in the Db group (*P* < 0.05) in post-test (month 4). (Table 3).

### The effects of T2D and HIIT on BALF cytokines and lung tissue oxidative stress

**Fig. 2:**
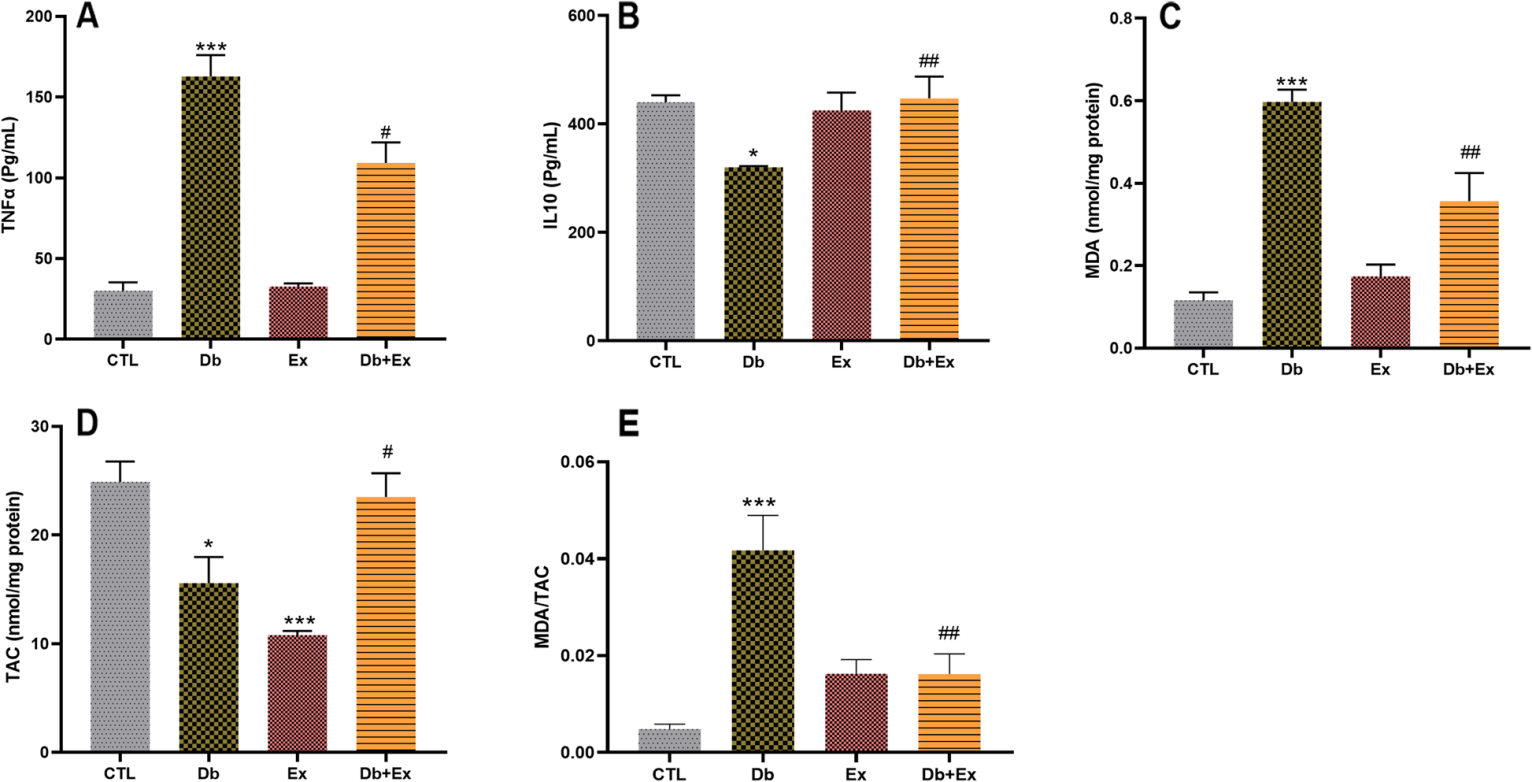
The effects of HIIT on BALF TNFα and IL-10 and lung tissue MDA, TAC and MDA/TAC ratio following T2D. The results are presented as mean ± SEM. CTL: control, Db: type 2 diabetic (STZ injected), Ex: exercise only, and Db + Ex: type 2 diabetic exercise. **A** The levels of tumor necrosis factor (TNFα). **B** The levels of interleukine-10 (IL-10). **C** Malondialdehyde (MDA), (**D**) Total anti-oxidant capacity (TAC), (**E**) MDA/TAC ratio. * *P* < 0.05 & *** *P* < 0.001 compared to the CTL and # *P* < 0.05 &## *P* < 0.01 compared to the Db

 BALF cytokines were evaluated to investigate the effects of HIIT on inflammation due to T2D. In the rats with T2D, the levels of TNF-α increased compared to the control group (*P* < 0.001). However, in Db + Ex group the levels of TNF-α were reduced (*P* < 0.05 vs. Db group) (Fig. 2A). The levels of IL-10 in the rats with T2D were decreased compared to the control group (*P* < 0.05), however in the Db + Ex group, the value of this cytokine elevated in comparison with the T2D group (*P* < 0.01) (Fig. 2B). Results showed that there was no significant difference between the exercise and control groups in relation to these cytokines.

T2D significantly increased the MDA level (*P* < 0.001) (Fig. 2C) and diminished the TAC level in lung tissue (*P* < 0.05) (Fig. 2D) and increased the MDA/TAC ratio (*P* < 0.001) (Fig. 2E) in comparison with the CTL group. Performing HIIT by Db + Ex group for 8 weeks significantly restored these alterations and the MDA/TAC ratio decreased in this group (*P* < 0.01 vs. Db group) so that it had not significant difference with CTL group (Fig. 2E). Despite the reduction of TAC in exercise group vs. CTL group (*P* < 0.001), MDA/TAC ratio didn’t significant difference between these groups.

### The effects of T2D and HIIT on apoptosis indices and surfactant proteins in BALF

**Fig. 3:**
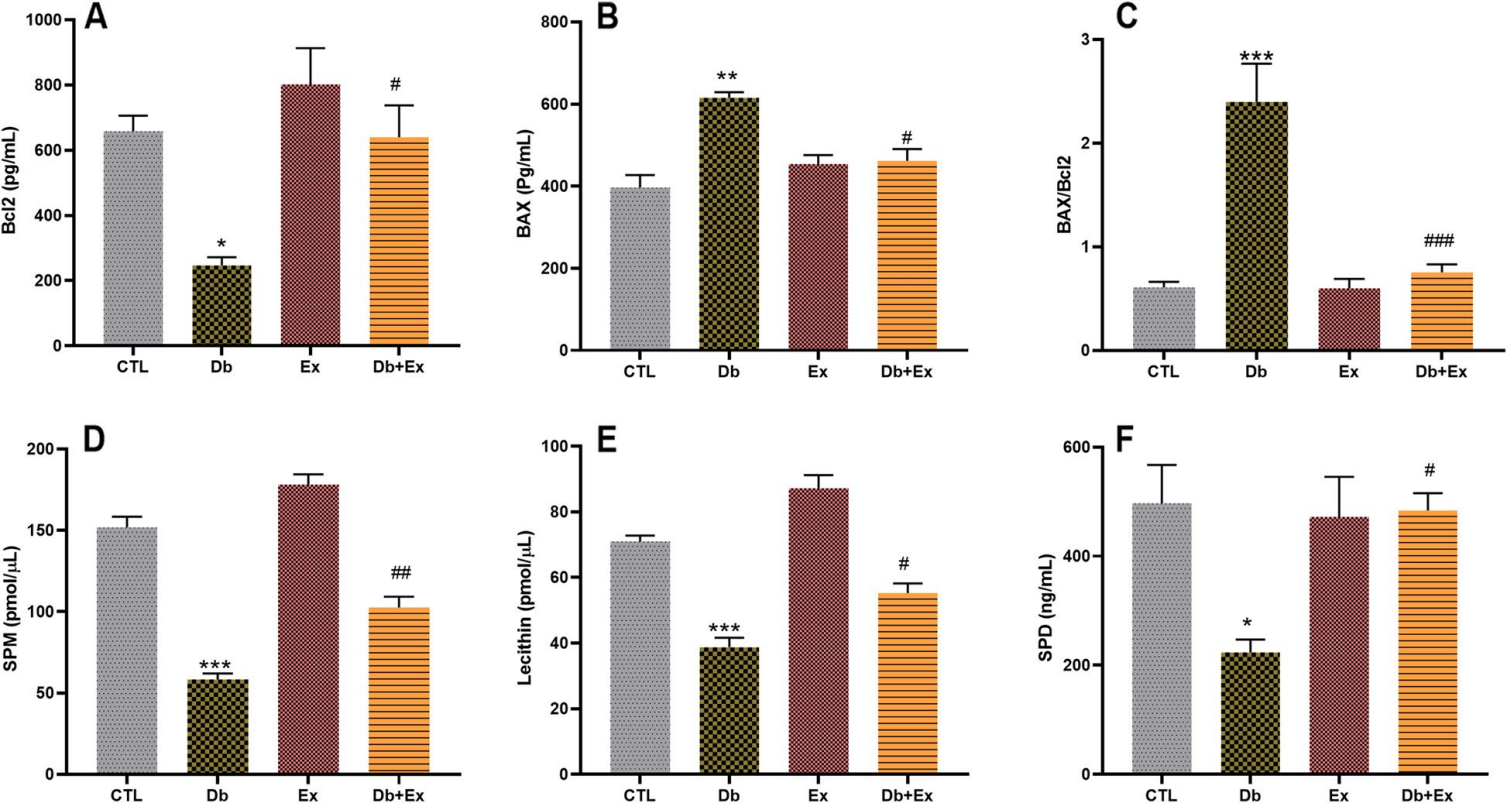
The effects of HIIT on BALF BAX, Bcl2 and their ratio and surfactant proteins following T2D. The results are presented as mean ± SEM. CTL: control, Db: type 2 diabetic (STZ injected), Ex: exercise only, and Db + Ex: type 2 diabetic + exercise. **A** Bcl2, (**B**) BAX, (**C**) BAX/Bcl2 ratio. **D **Sphingomyelin (SPM), (**E**) Lecithin, (**F**) Surfactant protein D (SPD). * *P* < 0.05 & ** *P* < 0.01& *** *P* < 0.001 compared to the CTL and # *P* < 0.05 & ## *P* < 0.01 & ### *P* < 0.001 compared to the Db

Our results revealed that the levels of Bcl2 in BALF of diabetic rats were decreased (*P* < 0.05) (Fig. 3A) while the levels of BAX were increased (*P* < 0.01) (Fig. 3B). Also, the BAX to Bcl2 ratio as an index of apoptosis/survival was higher in Db group than CTL group (*P* < 0.001) (Fig. 3C). Furthermore, 8 weeks of HIIT could improve apoptosis/survival index through increasing Bcl2 and reducing BAX compared to the Db group (*P* < 0.001). Our results showed that the status of apoptotic factors in the exercise group had not difference in comparison with the control group.

Our findings disclosed that the levels of surfactant components (SP-D, SPM and Lecithin) in Db group was lower than CTL group (*P* < 0.001 for SPM and lecithin & *P* < 0.05 for SPD) while following HIIT, the levels of these proteins were increased compared to the Db group significantly (*P* < 0.01 for SPM & *P* < 0.05 for lecithin and SPD) (Fig. 3D, E, F). There was no significant difference between exercise and control groups regarding pulmonary surfactants.

### The effects of T2D and HIIT and on lung fibrosis

**Fig. 4:**
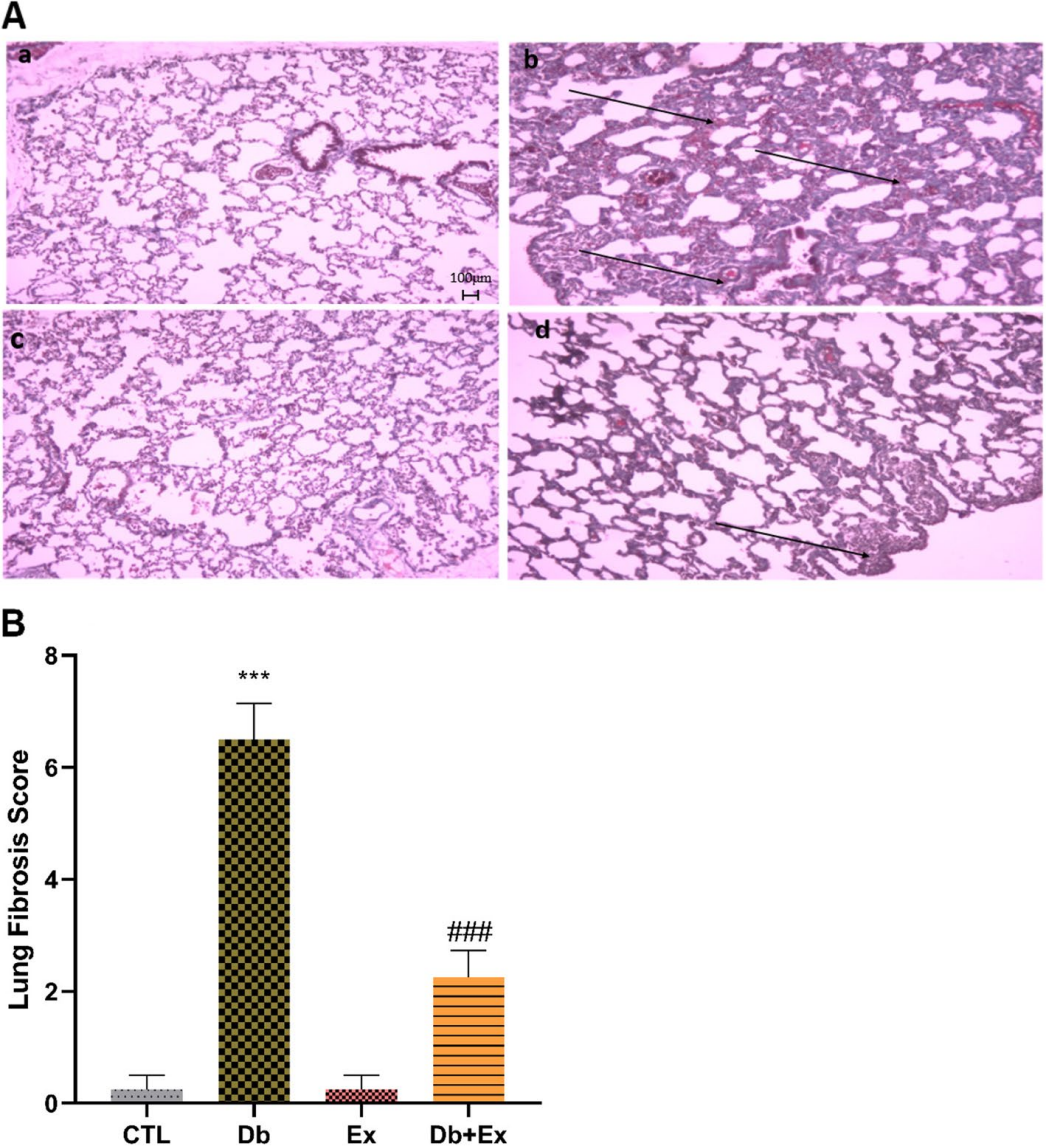
Micrographs of the lung (A) in CTL (a), Db (b); Ex (c); Db + Ex (d) groups. Comparison of fibrosis score in the lung tissue of different groups illustrated in figure (B). (Magnifcation:×40, scale bar: 40 μm).Black arrows indicate fibrotic areas. Data are means ± SEM .*** *P* < 0.001 compared to the CTL.### *P* < 0.001 compared to the Db

Fibrotic tissue was significantly increased in the lung of diabetic rats (*P* < 0.001), which mitigated following HIIT (*P* < 0.001) (Fig. 4A (micrographs) and Fig. 4B).

### The effects of T2D and HIIT on histological score and mean alveolar number

**Fig. 5:**
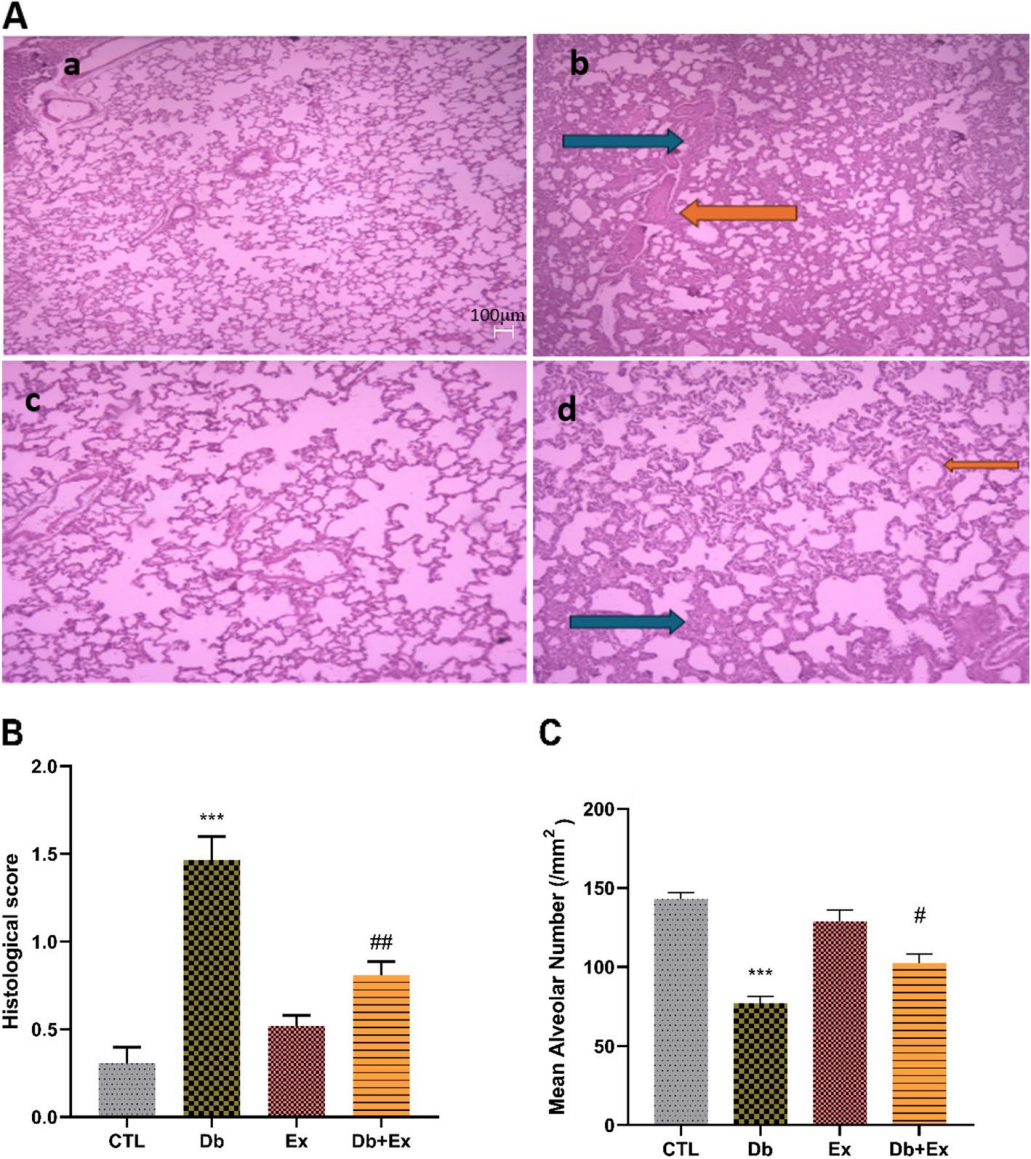
Micrographs of the lung (A) in CTL (a), Db (b); Ex (c); Db + Ex (d) groups. Comparison of histological score (B) and mean alveolar number (MAN) (C) in the lung tissue of different groups illustrated in this figure. (Magnifcation:×40, scale bar: 40 μm).Blue arrows indicate inflammation and orange arrows indicate congestion. Data are means ± SEM .*** *P* < 0.001 compared to the CTL. # *P* < 0.05 & ## *P* < 0.01 compared to the Db

Semi-quantitative histological scoring of the lungs demonstrated moderate to severe inflammation, interstitial leukocytes infiltration, congestion, destruction of septum of alveoli, expansion of the alveolar interstitial space, and fibrosis in diabetes group in comparison with CTL and Ex groups (*P* < 0.001). Combination of exercise with diabetes significantly decreased the negative effect of diabetes (*P* < 0.01); however, there was still some degree of damage in Db + Ex group (Fig. 5A(c) and B). Diabetes was associated with reduction in AN/SA (mm^2^) when compared with CTL and Ex groups (Fig. 5A (d) and C) (*P* < 0.001) and exercise training attenuated this effect (*P* < 0.05) (Fig. 5A (c) and C).

## Discussion

The aim of this study was the examination of the effects of 8 weeks of HIIT on tissue injury, some pro/anti-inflammatory cytokines, survival/apoptotic proteins, redox balance and surfactant components in lung of diabetic rats.

Results showed that following T2D, inflammation, oxidative stress, apoptosis, and injury increased in lung tissue and BALF. Also, the levels of pulmonary surfactants components in BALF were reduced due to T2D.On the other hand, 8 weeks of HIIT could reverse all mentioned alterations toward normal levels.

The trends of FBG and BW changes (Table 3) approved the T2D induction and were in line with other previous studies [25, 35, 36] .Also, we showed the serum insulin reduction in T2D rats in our previous research [35].In addition, as reported in our previous publications, food intake was increased owing to T2D (polyphagia) and we demonstrated that HIIT may modulate appetite regulation in rats with T2D through leptin signaling [35].

The lung has a complex alveolar-capillary network which may be targeted by diabetic damage [37]. Diabetic patients frequently report respiratory symptoms [38] and are at increased risk of several pulmonary diseases [39]. It has been shown that T2D is associated with an increased prevalence of respiratory symptoms as compared to the general population [38].

Present study revealed that diabetes increased TNFα as a pro-inflammatory cytokine and reduced IL-10 as an anti-inflammatory cytokine in BALF (Fig. 2A & B). Consistent with our findings, Talakatta et al. showed increasing levels of TNFα and decreasing levels of IL10 in the serum of diabetic animals [40]. Also, Dennis et al. reported that increased TNFα levels are associated with inadequate glucose control in T2D and impaired lung function [41]. In another study, it has been shown that the levels of IL-6 and IL-17 were increased in diabetic lungs [42]. Diabetes is a pro-inflammatory state associated with airway inflammation [43]. Hyperglycemia increases chronic inflammation, inflammatory cytokine release, and oxidative stress through activation of the nuclear factor kappa B (NFκB) pathway and NADPH oxidase (NOX) as well as the production of reactive oxygen species (ROS) and reactive nitrogen species (RNS) [44]. Our findings showed that HIIT could ameliorate inflammation through increasing IL10 and decreasing TNFα in diabetic rats. Consistent with this, some investigations have disclosed the important role of HIIT in reducing inflammation [45–47]. Mohammadi zadeh et al. showed that HIIT reduces pro-inflammatory markers and increases anti-inflammatory markers in T2D patients [48].In addition, Azizi et al. demonstrated that swimming training reduced inflammation in pulmonary tissue through diminishing IL1β in type 1 diabetic mice [49]. Thus, it seems that HIIT has anti-inflammatory effects in diabetic lung.

The other findings of the present study were decreasing levels of TAC and increasing levels of MDA and MDA/TAC as an index of redox imbalance in favor of oxidant components in lung tissue following T2D (Fig. 2C-E). Performing 8 weeks HIIT by diabetic rats improved the redox balance in our study. In line with these findings, evidence indicates that in the diabetic lung, the activity of superoxide dismutase (SOD) was decreased, while the contents of nitric oxide (NO) and malondialdehyde (MDA) were significantly increased [50]. MDA has been documented as a primary biomarker of free radical mediated lipid damage and oxidative stress [51]. Increased level of MDA in diabetics suggests that peroxidative injury may be involved in the development of diabetic complications. The increase in lipid peroxidation is also an indication of decline in defense mechanisms of enzymatic and nonenzymatic antioxidants [52].On the other hand, it has been shown that the TAC level was reduced following T2D [53]. The positive effect of HIIT on reducing oxidative stress has been shown in various tissues by decreasing lipid peroxidation and enhancing antioxidants defenses [54–56]. Also, Machado et al. revealed that treadmill running (5 days a week for 9 weeks) increased SOD and catalase in lung of newborn diabetic rats [57].The reduction of MDA/TAC ratio as an index of oxidant/anti-oxidant index following HIIT confirms the positive effect of this type of training and is in line with previous findings.

Our findings demonstrated the apoptosis activity as decreasing level of Bcl2 and increasing level of BAX and BAX/Bcl2 ratio in lung of diabetic rats and HIIT reversed this process (Fig. 3A-C). It has been shown that apoptosis of lung epithelial cells was increased in diabetic animals compared to non-diabetic. This was associated with increased inflammation and oxidative stress in the lungs of diabetic rats [58]. High blood sugar levels in diabetic rats leads to increased apoptosis of lung cells and decreased lung function [59]. BAX and Bcl2 may play a role in the pathogenesis of diabetic lung disease. For example, one study found that BAX expression was increased in the lung tissue of diabetic rats while the Bcl2 expression was decreased and that this was associated with increased apoptosis and lung injury [60]. Consistent with our finding, previous studies indicated that HIIT reduced apoptosis via increasing Bcl2 and decreasing BAX expression in heart following diabetes [61, 62]. Also, it has been shown that swimming exercise has anti-apoptotic impacts through reducing BAX in lungs of diabetic mice [49].

Our results revealed that the levels of SP-D, SPM and lecithin diminished in BALF of diabetic rats while HIIT could improve these components (Fig. 3D-F). One study disclosed that diabetic rats had decreased levels of SP-A and SP-B, which are important components of the pulmonary surfactant system [63]. SP-D is an important regulatory protein that may aid in controlling chronic inflammation, reducing oxidative radical formation, facilitating phagocytosis and agglutination, reducing cell death, and enhancing apoptotic and necrotic cell clearance and it has been shown that SP-D reduce due to T2D [64]. Lopez et al. demonstrated that Serum SP-D concentration can be a useful biomarker for detecting lung impairment in obese patients with T2D [65]. The effect of exercise on the level of pulmonary surfactants following diabetes has not been well investigated. However, Increment of pulmonary surfactant (surfactant protein A) of young male rats after six weeks interval training showed by Mirdar et al. [66]. Another study revealed that exercise can improve pulmonary surfactants due to lung injury [67]. The present study suggests that HIIT may have a beneficial role in maintaining pulmonary function by restoring lung surfactant components in diabetes.

We observed increased fibrotic tissue and obvious pathoβlogical changes in lung tissue due to T2D, and HIIT significantly improved these malformations (Figs. 4 and 5). In line with this finding, it has been shown that hyperglycemia in diabetes accelerates fibrotic changes in the lung through the activation of TGF-β signaling pathways [40]. Also, Machado et al [57] showed pathological changes such as increased bronchoconstriction index and polymorphonuclear cells in the lungs of diabetic rats that recovered by moderate exercise training. Before all the effects, we indicated that HIIT was able to lower blood sugar in diabetic rats (Table 3). It has been shown that HIIT as a time-efficient exercise option can be safe and effective for reducing blood glucose levels in individuals with, or at risk for, T2D [68]. Little et al. showed that Low-volume HIIT reduces hyperglycemia in patients with T2D [69]. Therefore, HIIT can be useful in improving pulmonary lesions following T2D by reducing blood sugar levels.

## Limitations and future perspectives

It is mentioned that in this study, we examined and measured some anti-inflammatory cytokines, total anti-oxidant capacity, two important proteins involved in apoptosis, and surfactant proteins.However, due to finance limitations, we were not able to measure other factors such as other pro-inflammatory interleukines such as IL-6, IL-4, IL1β and IL-17 and the activity of MPO, GPX, SOD and caspases involved in apoptosis, which complement and strengthen the present findings of this study.The measurment of the above mentioned items will be considered in future studies.

## Conclusion

The findings of the present study showed that, 8 weeks of HIIT as a non-pharmacological intervention can improve inflammation, oxidative stress, apoptosis, and histopathological changes in the lungs of diabetic rats. Also, HIIT can increase the level of surfactant components in the BALF of diabetic rats and hence may contribute to the improvement of pulmonary function. It seems that these positive effects of HIIT are caused by its anti-hyperglycemic effects thereby maintaining the balance of redox, apoptosis, and inflammatory/anti-inflammatory systems. Exercise is a non-pharmacological intervention and has few side effects compared to drug treatments. Considering the promising results of this study and similar animal studies on the protective effects of exercise against diabetic lung, more clinical studies are suggested to generalize these findings to humans.

### Supplementary Information

**Additional file 1.**

## Data Availability

The datasets used and/or analyzed during the current study are available from the corresponding author on reasonable request.
